# A Theoretical Analysis of Why Hybrid Ensembles Work

**DOI:** 10.1155/2017/1930702

**Published:** 2017-01-31

**Authors:** Kuo-Wei Hsu

**Affiliations:** Department of Computer Science, National Chengchi University, No. 64, Sec. 2, Zhi Nan Rd., Wen Shan District, Taipei City 11605, Taiwan

## Abstract

Inspired by the group decision making process, ensembles or combinations of classifiers have been found favorable in a wide variety of application domains. Some researchers propose to use the mixture of two different types of classification algorithms to create a hybrid ensemble. Why does such an ensemble work? The question remains. Following the concept of diversity, which is one of the fundamental elements of the success of ensembles, we conduct a theoretical analysis of why hybrid ensembles work, connecting using different algorithms to accuracy gain. We also conduct experiments on classification performance of hybrid ensembles of classifiers created by decision tree and naïve Bayes classification algorithms, each of which is a top data mining algorithm and often used to create non-hybrid ensembles. Therefore, through this paper, we provide a complement to the theoretical foundation of creating and using hybrid ensembles.

## 1. Introduction

Ensemble learning is inspired by the human group decision making process and has gained much attention [1–7]. It is to create an ensemble, which is a group of classifiers and combines classifications made by these classifiers to make an overall classification. The advantage of ensembles is not that the best combination of classifiers outperforms the best classifier but that a combination of classifiers is less probable to misclassify unseen data samples than a single classifier. Ensembles have shown their satisfactory classification performance in a large scale comparative study [8].

Ensemble learning has been applied in various application domains, such as image classification [9–14], fingerprint classification [15], weather forecasting [16], text categorization [17], image segmentation [18], visual tracking [19], protein fold pattern recognition [20], cancer classification [21], pedestrian recognition [22] or detection [23], prediction of software quality [24, 25], face recognition [26], email filtering [27], prediction of students' performance [28], medical image analysis [29–32], churn prediction [33], sentiment analysis [34–37], steganalysis [38], prediction of air quality [39], and intrusion detection [40].

From one point of view, ensemble learning becomes popular because every classification algorithm has its own limitations. From another point of view, if every classifier in an ensemble has expertise in classifying data samples that belong to some portion of the given data set, the overall classification combined from all (or some) classifiers in the ensemble will potentially be more reliable.

Compared to classifiers working individually, classifiers working together will have a better potential for gaining better accuracy [41]. Diverse classifiers working together will have a better potential for gaining better accuracy compared to non-diverse classifiers working together, as suggested in [42]. In creating an ensemble, every classifier that will be part of the ensemble is expected to be adequately accurate, while the correlation between classifications made by two classifiers that will be part of the ensemble is expected to be small.

Some researchers use a mixture of different types of classification algorithms to create hybrid ensembles. For example, with reference to ensembles composed of decision trees and artificial neural networks, Langdon et al. use such an ensemble in drug discovery [43]; Lu et al. discuss sampling methods along with these ensembles for active learning [44]; in [45], the author analyzes the hybrid ensembles for binary classification from the standpoint of bias-variance decomposition proposed in [46]. Furthermore, Salgado et al. use ensembles of artificial neural networks and support vector machines [47, 48] to predict daily electricity load [49]. Min and Cho use naïve Bayes classifiers and support vector machines for activity recognition [50]. Verikas et al. provide a survey of hybrid ensembles designed for bankruptcy prediction [51]. In addition, Verma and Hassan use integrations of clustering and classification algorithms to create hybrid ensembles [52].

Most researchers simply use hybrid ensembles without further investigation. Therefore, we plan to have a better understanding of hybrid ensembles. Our goal is not only to show that the classification performance of hybrid ensembles can be comparable or even superior to that of non-hybrid ensembles, but also to provide an explanation of why hybrid ensembles work from the standpoint of diversity. Diversity among classifiers in an ensemble plays a significant role in the success of the ensemble. One type of ensemble learning algorithms uses sampling methods to generate different data sets used for training diverse classifiers, such as Bagging (Bootstrap Aggregating) [53], while another type is rather ad hoc and uses different classification algorithms to train diverse classifiers that will be used to create an ensemble.

We create hybrid ensembles by using an integration of these two types of ensemble learning algorithms. The classification algorithms that we use to create hybrid ensembles are C4.5 [48, 54] decision tree and naïve Bayes [48]. We provide an empirical comparison of these hybrid ensembles and others created by using Bagging. This paper is particularly essential because quite few papers empirically evaluate hybrid ensembles and at the same time theoretically analyze them.

The rest of this paper is structured as follows: We provide background information and present our analysis in Section 2. Next, we report and discuss experiment results in Section 3. Finally, we give conclusion in Section 4.

## 2. Materials and Methods

The goal of using several classifiers in an ensemble is to achieve better classification performance by combining classifications from these classifiers, each of which serves as an optimal model or an expert in a portion of the data set. These classifiers are expected to be uncorrelated and behave independently of each other; or, at least, they need to show different patterns of errors.

Diversity among classifiers in an ensemble is related to the success of the ensemble, because it can compensate for errors made by those classifiers individually [55]. In this section, we analyze diversity and classification performance, for example, accuracy, of ensembles created by using a single algorithm (i.e., non-hybrid ensembles) and hybrid ensembles created by using two different algorithms. The relationship between diversity and accuracy is not* “straightforward”* [56]. On one hand, combining classifications from classifiers of low diversity would not improve the overall accuracy because these classifiers behave similarly for some portions of the data set; on the other hand, using highly diverse classifiers to create an ensemble would not guarantee absolutely high accuracy. Hsu and Srivastava show that using different classification algorithms in an ensemble would likely increase diversity and decrease correlation between classifiers in the ensemble [57], and they build the connection between diversity and correlation, which can be indirectly connected to accuracy [58]. Referring to the analysis technique used in [59], we build a more direct connection between diversity and accuracy, and further we analyze the influence of using different algorithms to create an ensemble on accuracy; the analysis distinguishes this paper from the earlier papers.

In what follows, *x* is a *d*-dimensional vector to represent a data sample, and *y* is a binary class label or *y* ∈ {−1, 1}. *C* is a classifier and $C:x\in {\mathbb{R}}^{d}\mapsto \hat{y}\in \left\{-1,\text{1}\right\}$, where $\hat{y}$ is a classification made by a classifier. *A* is a classification algorithm. *T* is a set of data samples. *C*(*x*∣*A*, *T*) means that *C* is trained by applying *A* on *T* and is used to classify *x*, and it returns a binary class label.

What is described in (1) is a general form of an ensemble where classifications from classifiers are combined through majority voting to make the overall classification. It can be modified such that it uses weighted majority voting. In (1), *C* is an ensemble of  *n* classifiers, *C*_*i*_ is a classifier in the ensemble, *x* is a data sample that needs to be classified, and *v* is the overall classification. For those ensemble learning algorithms only using different data sets to train diverse classifiers, *A*_*i*_ = *A*_*j*_ and *T*_*i*_ ≠ *T*_*j*_ for all *i* ≠ *j*, where 0 ≤ *i*, *j* ≤ *n*, and Bagging is an example. For those using different classification algorithms and different data sets to train diverse classifiers, *A*_*i*_ = *A*_*j*_ for some (or, in few cases, all) *i* ≠ *j* and *T*_*i*_ ≠ *T*_*j*_ for all *i* ≠ *j*, where 0 ≤ *i*, *j* ≤ *n*, and this is of our interest in this paper

(1) Cxarg maxv=−1,1∑i=1nIv=y^i=arg maxv=−1,1∑i=1nIv=Cix ∣ Ai,Ti,where  I  is  the  indicator  function.

Given a data set *D* and a set of classification algorithms *G*, we create a hybrid ensemble of *n* classifiers, each of which is trained by applying an algorithm selected in an alternating fashion from *G* on a set of data samples drawn from *D* with bootstrap sampling. This process is shown in Algorithm 1. Instead of selecting algorithms in an alternating fashion, we can simply do random selection such that a classifier in the hybrid ensemble could be trained by using one of the algorithms in *G* with an equal probability. Extending from this, we can incorporate prior knowledge and assign unequal probabilities to different algorithms.

The input of the bootstrap sampling method is a data set *D*, and the output is a set *D*_*b*_ of data samples drawn with replacement from *D*, |*D*_*b*_ | = |*D*|. We use bootstrap sampling when we need different data sets to train diverse classifiers in creating an ensemble. Diversity among the classifiers in such an ensemble totally comes from differences among the data sets used to train the classifiers, and therefore bootstrap sampling is the single generator of diversity in such an ensemble. We train diverse classifiers by additionally using different classification algorithms, and by doing this we have an additional generator of diversity.

Afterward, we present our analysis of why hybrid ensembles work based on the analysis technique used in [59]. We start from the following definitions, where ${\hat{y}}^{\left(A,T\right)}=C\left(x|A,T\right)$ and $\hat{y}$ is the classification to a testing data sample *x* given by a classifier *C* trained by applying *A* on *T*.

**Definition 1 .**

*D* is a given data set and *W* is a set of data sets generated with bootstrap sampling from *D*. The *i*th element in *W* is of the same size of *D*; that is, |*W*_*i*_ | = |*D*|. *T*_1_ ∈ *W* and *T*_2_ ∈ *W* are used as training data sets, where *T*_1_ ≠ *T*_2_. With respect to *S* ∈ *W* used as a testing data set, where *S* ≠ *T*_1_ and *S* ≠ *T*_2_, *T*-Diversity of a classification algorithm *A* is defined as an expectation of disagreement between classifiers trained by applying *A* on *T*_1_ and *T*_2_, as given in

(2) T-Diversity=ESIy^A,T1≠y^A,T2,where  ES  is  the  expectation  upon  S.

Definition 1 describes how unstable a classification algorithm could possibly be on a given data set.  Definition 2 describes the degree of being unstable (or the instability) for a classification algorithm.

**Definition 2 .**

Based on Definition 1, a classification algorithm *A* is (*α*, *β*)-unstable with respect to *T*-Diversity, if the following holds:

(3) PWESIy^A,T1≠y^A,T2≥α≥β,where  PW  is  the  probability  upon  W.

Here, disagreement between classifiers is from using different training data sets, and it is a type of diversity. In (3), *β* is the lower bound of the probability that we can observe disagreement at least *α*; the larger the *α*, the larger the diversity. When *α* is treated as a constant, a larger value of *β* means a more unstable classification algorithm.

We use the process given below to estimate the instability of an algorithm on a data set. The idea is to use two sampled data sets to train two classifiers and then measure the difference in classifications made by the two classifiers on another sampled data set. Because these data sets are from the same given data set, difference in classifications mainly comes from the algorithm used to train the classifiers. If the algorithm is more unstable and sensitive to the changes in the data sets used for training, the trained classifiers would make more different classifications on a data set, part of which has been seen by both classifiers. The larger the value returned by the process, the more unstable the algorithm.

**Definition 3 .**

*D* is a given data set and *W* is a set of data sets generated with bootstrap sampling from *D*. The *i*th element in *W* is of the same size of *D*; that is, |*W*_*i*_ | = |*D*|. *T* ∈ *W* is used as a training data set. With respect to *S* ∈ *W* used as a testing data set, where *S* ≠ *T*, *A*-Diversity for two classification algorithm *A*_1_ and *A*_2_ is defined as an expectation of disagreement between classifiers trained by applying *A*_1_ and *A*_2_ on *T*, as given in

(4) A-Diversity=ESIy^A1,T≠y^A2,T,where  ES  is  the  expectation  upon  S.

Definition 3 describes how different two classification algorithms could possibly be on a given data set.  Definition 4 describes the degree of being different (or the differentiability) for two classification algorithms.

**Definition 4 .**

Based on Definition 3, two classification algorithms *A*_1_ and *A*_2_ are (*δ*, *γ*)-differentiable with respect to *A*-Diversity, if the following holds:

(5) PWESIy^A1,T≠y^A2,T≥δ≥γ,where  PW  is  the  probability  upon  W.

Here, disagreement between classifiers is from using different classification algorithms, and it is a type of diversity, too. In (5), *γ* is the lower bound of the probability that we can observe disagreement at least *δ*; the larger the *δ*, the larger the diversity. Similarly, when *δ* is treated as a constant, a larger value of *γ* means that two classification algorithms are more different.

We use the process given below to estimate the differentiability of a pair of algorithms on a data set. The idea is to use two algorithms and a sampled data set to train two classifiers and then measure the difference in classifications made by the two classifiers on another sampled data set. If the two algorithms are more differentiable, the classifiers trained with them would make more different classifications on a data set, part of which has been seen by both classifiers. The larger the value returned by the process, the more differentiable the two algorithms.

We show a connection between diversity and accuracy gain for using a hybrid ensemble through the proposition given below.

**Proposition 1 .**

If two classification algorithms *A*_1_ and *A*_2_ are (*δ*, *γ*)-differentiable and *A*_1_ is (*α*_1_, *β*_1_)-unstable, then (6) holds, where Δ*E*[*Acc*] is the difference between the expected accuracy of a hybrid ensemble with *A*_1_ and *A*_2_ and the expected accuracy of a non-hybrid ensemble with only *A*_1_, and *Acc*_*i*_ is the accuracy of the classifier training by using *A*_*i*_

(6) PΔEAcc≥α1·δ·Acc2−Acc1≥1−1−β1·1−γ.

Regarding (6), *A*_1_ is the classification algorithm used to train most classifiers in both ensembles, and *A*_2_ is the classification algorithm used to train a classifier in a hybrid ensemble. When *α*_1_ and *δ* are treated as constants, the lower bound of the probability that we can observe accuracy gain depends on how unstable *A*_1_ is and how different *A*_1_ and *A*_2_ are. If *β*_1_ is larger, meaning that *A*_1_ is more unstable, and *γ* is larger, meaning that *A*_1_ and *A*_2_ are more different, then the lower bound of the probability is larger; if *β*_1_ is smaller, meaning that *A*_1_ is more stable, and *γ* is smaller, meaning that *A*_1_ and *A*_2_ are more similar, then the lower bound of the probability is smaller, while this does not necessarily mean that the probability of observing accuracy gain is smaller. As a result, if the algorithm used to train most classifiers in a hybrid ensemble is unstable and it is different from the other algorithm, then it is more probable that accuracy gain would be observed. Furthermore, the lower bound of the accuracy gain depends on how unstable *A*_1_ is, how different *A*_1_ and *A*_2_ are, and how accurate the classifiers trained with *A*_1_ and *A*_2_ are. If the classifier trained with *A*_2_ is more accurate, *Acc*_2_ − *Acc*_1_ is larger and the lower bound of accuracy gain is larger. As a result, it is beneficial to replace a classifier (or some classifiers) in a non-hybrid ensemble with a classifier (or some classifiers) trained with a different yet accurate classification algorithm. Nevertheless, it may not be beneficial to replace all, because doing so would lower diversity among classifiers.

**Proof**

*T*_1_, *T*_2_, and *T*_3_ are three data sets generated with bootstrap sampling from a given data set. Classifiers *C*_1_, *C*_2_, and *C*_3_ are trained by applying a classification algorithm *A*_1_ on *T*_1_, *T*_2_, and *T*_3_, respectively. That is, $C_{1}(x|A_{1},T_{1})={\hat{y}}^{\left(A_{1},T_{1}\right)}$, $C_{2}(x|A_{1},T_{2})={\hat{y}}^{\left(A_{1},T_{2}\right)}$, and $C_{3}\left(x|A_{1},T_{3}\right)={\hat{y}}^{\left(A_{1},T_{3}\right)}$. The ensemble composed of *C*_1_, *C*_2_, and *C*_3_ is a non-hybrid ensemble, because it is with only a classification algorithm. Classifier *C*_2_′ is trained by applying another classification algorithm *A*_2_ on *T*_2_. That is, $C_{2}^{\prime }\left(x|A_{2},T_{2}\right)={\hat{y}}^{\left(A_{2},T_{2}\right)}$. The ensemble composed of *C*_1_, *C*_2_′, and *C*_3_ is a hybrid ensemble, because it is with two different classification algorithms.

Initially, we represent the expected accuracy of the non-hybrid ensemble in (7), which is based on majority voting. In what follows, *y* is the class label of a data sample

(7) EIy^A1,T1=y∧y^A1,T2=y∧y^A1,T3=y+Iy^A1,T1=y∧y^A1,T2=y∧y^A1,T3≠y+Iy^A1,T1=y∧y^A1,T2≠y∧y^A1,T3=y+Iy^A1,T1≠y∧y^A1,T2=y∧y^A1,T3=y.

Similarly, we represent the expected accuracy of the hybrid ensemble in

(8) EIy^A1,T1=y∧y^A2,T2=y∧y^A1,T3=y+Iy^A1,T1=y∧y^A2,T2=y∧y^A1,T3≠y+Iy^A1,T1=y∧y^A2,T2≠y∧y^A1,T3=y+Iy^A1,T1≠y∧y^A2,T2=y∧y^A1,T3=y.

The difference between the expected accuracy of the hybrid ensemble and that of the non-hybrid ensemble or the difference between (8) and (7) is denoted by Δ*E*[Acc]. It is given in

(9) ΔEAcc=EIy^A1,T1=y∧y^A2,T2=y∧y^A1,T3=y+Iy^A1,T1=y∧y^A2,T2=y∧y^A1,T3≠y+Iy^A1,T1=y∧y^A2,T2≠y∧y^A1,T3=y+Iy^A1,T1≠y∧y^A2,T2=y∧y^A1,T3=y−Iy^A1,T1=y∧y^A2,T2=y∧y^A1,T3=y−Iy^A1,T1=y∧y^A2,T2=y∧y^A1,T3≠y−Iy^A1,T1=y∧y^A2,T2≠y∧y^A1,T3=y−Iy^A1,T1≠y∧y^A2,T2=y∧y^A1,T3=y.

Next, (9) is rewritten as (10) by rearranging its components

(10) ΔEAcc=EIy^A1,T1=y·Iy^A1,T3=y×Iy^A2,T2=y−Iy^A1,T2=y+Iy^A1,T1=y·Iy^A1,T3≠y×Iy^A2,T2=y−Iy^A1,T2=y+Iy^A1,T1=y·Iy^A1,T3=y×Iy^A2,T2≠y−Iy^A1,T2≠y+Iy^A1,T1≠y·Iy^A1,T3=y×Iy^A2,T2=y−Iy^A1,T2=y.

$𝕀\left({\hat{y}}^{\left(A_{2},T_{2}\right)}\neq y\right)-𝕀\left({\hat{y}}^{\left(A_{1},T_{2}\right)}\neq y\right)$ is equal to $\left(1-𝕀\left({\hat{y}}^{\left(A_{2},T_{2}\right)}=y\right)\right)-\left(1-𝕀\left({\hat{y}}^{\left(A_{1},T_{2}\right)}=y\right)\right)$, and further the component is equal to $-1\cdot \left(𝕀\left({\hat{y}}^{\left(A_{2},T_{2}\right)}=y-𝕀\left({\hat{y}}^{\left(A_{1},T_{2}\right)}=y\right)\right)\right)$. Furthermore, $𝕀\left({\hat{y}}^{\left(A_{1},T_{1}\right)}=y\right)\cdot 𝕀\left({\hat{y}}^{\left(A_{1},T_{3}\right)}\neq y\right)$ is equal to $𝕀\left({\hat{y}}^{\left(A_{1},T_{1}\right)}=y\wedge {\hat{y}}^{\left(A_{1},T_{3}\right)}\neq y\right)$, and the relationship ${\hat{y}}^{\left(A_{1},T_{1}\right)}=y\wedge {\hat{y}}^{\left(A_{1},T_{3}\right)}\neq y$ implies ${\hat{y}}^{\left(A_{1},T_{1}\right)}\neq {\hat{y}}^{\left(A_{1},T_{3}\right)}$. $𝕀\left({\hat{y}}^{\left(A_{1},T_{1}\right)}\neq y\right)\cdot 𝕀\left({\hat{y}}^{\left(A_{1},T_{3}\right)}=y\right)$ is equal to $𝕀\left({\hat{y}}^{\left(A_{1},T_{1}\right)}\neq y\wedge {\hat{y}}^{\left(A_{1},T_{3}\right)}=y\right)$, and the relationship ${\hat{y}}^{\left(A_{1},T_{1}\right)}\neq y\wedge {\hat{y}}^{\left(A_{1},T_{3}\right)}=y$ implies ${\hat{y}}^{\left(A_{1},T_{1}\right)}\neq {\hat{y}}^{\left(A_{1},T_{3}\right)}$, too. Next, (11) is obtained:

(11) ΔEAcc=EIy^A1,T1≠y^A1,T3×Iy^A2,T2=y−Iy^A1,T2=y.

In (11), the first component is related to *T*-Diversity and the second component is related to accuracy. As the two components are independent, (11) is rewritten as

(12) ΔEAcc=EIy^A1,T1≠y^A1,T3×EIy^A2,T2=y−Iy^A1,T2=y.

Next, by referring to Definition 4, the second component in (12) is associated with $P\left[{\hat{y}}^{\left(A_{1},T_{2}\right)}\neq {\hat{y}}^{\left(A_{2},T_{2}\right)}\wedge {\hat{y}}^{\left(A_{2},T_{2}\right)}=y\right]-P\left[{\hat{y}}^{\left(A_{1},T_{2}\right)}\neq {\hat{y}}^{\left(A_{2},T_{2}\right)}\wedge {\hat{y}}^{\left(A_{1},T_{2}\right)}=y\right]$, which is equal to

(13) $$\begin{array}{c} P\left[\;{\hat{y}}^{\left(A_{1},T_{2}\right)}\neq {\hat{y}}^{\left(A_{2},T_{2}\right)}\;\right]\cdot \left[\;{\text{Acc}}_{2}-{\text{Acc}}_{1}\;\right]. \end{array}$$

Because *A*_1_ and *A*_2_ are (*δ*, *γ*)-differentiable, (14) is larger than or equal to *δ* · [Acc_2_ − Acc_1_] with a probability at least *γ*. Combining this and *A*_1_ being (*α*_1_, *β*_1_)-unstable, (14) is obtained:

(14) PΔEAcc<α1·δ·Acc2−Acc1<1−β1·1−γ.

Finally, (15) is obtained through (14) and the proof is complete:

(15) PΔEAcc≥α1·δ·Acc2−Acc1≥1−1−β1·1−γ.

We treat *α*_1_ and *δ* as constants, as in the processes shown in Algorithms 2 and 3. Given an ensemble of classifiers trained by using *A*_1_. We replace some classifiers with those trained by using *A*_2_, which generally provides a higher value of accuracy (so that Acc_2_ is larger than Acc_1_), and the lower bound of accuracy gain will be positive, meaning that we would probably obtain a hybrid ensemble that could achieve better classification performance. If is *A*_1_ more unstable, *β*_1_ will be larger. If *A*_2_ is more different from *A*_1_, *γ* will be larger. The larger *β*_1_ and the larger *γ*, the larger the lower bound of the probability that we would observe better accuracy.

## 3. Results and Discussion

### 3.1. Data Sets

In experiments, we use 20 data sets from UCI Machine Learning Repository [60], supported by School of Information and Computer Science, University of California, Irvine, and from Department of Statistics, Carnegie Mellon University. Using public data sets is to allow the reproduction of the experiments.

The data sets used in experiments are from various application domains. Their characteristics are summarized in Table 1, where the first column is the serial number, the second column is the name of a data set, the third column is the number of data samples, the fourth column is the number of data samples that belong to the minority class, the fifth column is the number of nominal attributes, the sixth column is the number of numeric attributes, and the numbers in parentheses present the numbers of attributes with missing values. The proportion of minority samples affects the classification performance of a classifier or an ensemble, and so does the proportion of attributes with missing values. For a data set that is usually used in regression analysis, we apply discretization on its target attribute and divide continuous values of the attribute properly into two intervals each of which corresponds to a class label; such a data set is with the suffix “binary.”

### 3.2. Settings

We consider C4.5 decision tree and naïve Bayes classification algorithms. The former is denoted by DT, and the latter is denoted by NB. We consider single classifiers trained by using DT or NB, non-hybrid ensembles trained with DT or NB, and hybrid ensembles trained with DT and NB.

We implement the process given in Algorithm 1 by using WEKA [61], and we compare the classification performances given by ensembles created by it to that given by Bagging. The ensembles created by using Bagging with DT or NB are non-hybrid ensembles with only DT or NB. We set the number of classifiers in an ensemble to 10 for all ensemble learning algorithms. We use 20 × 10-fold cross-validation for classification performance evaluation. That is, for each combination of an ensemble learning algorithm and a data set, we run 10-fold cross-validation 20 times randomly and independently. In addition, we investigate instability and differentiability.

### 3.3. Instability

We use the process described earlier to estimate the instability of an algorithm on a data set. We set *α* to 0.05 (by statistical convention) and the number of trials to 100. We report the results in Table 2. The content in a cell indicates the value of *β* or the probability that the disagreement rate is larger than or equal to *α* or 0.05. The disagreements are given by two classifiers trained by applying the algorithm corresponding to the column on two data sets sampled from the data set corresponding to the row. The probability is calculated with reference to 100 trials. The larger the value of *β*, the more unstable the algorithm on the data set. DT is more unstable than NB on 13 out of these 20 data sets. This is consistent with the general thought that decision tree algorithm is unstable (or it is a classification algorithm with high variance) and therefore suitable for being used to create ensembles. The difference of values of instability is larger than or equal to 0.5 on 4 data sets:* heart-c-binary*,* heart-h-binary*,* hprice-binary*, and* sonar*. The results clearly show that instability results from not only the nature of a data set but also the nature of a classification algorithm.

### 3.4. Differentiability

We use the process described earlier to estimate the differentiability of two algorithms on a data set. We set *δ* to 0.05 (by statistical convention) and the number of trials to 100. We report the results in Table 3. The content in a cell indicates the value of *γ* or the probability that the disagreement rate is larger than or equal to *δ* or 0.05. The disagreements are given by two classifiers trained by applying DT and NB separately on a data set sampled from the data set corresponding to the row. The probability is calculated with reference to 100 trials. The larger the value of *γ*, the more different the two algorithms on the data set. According to Table 3, DT and NB are different or behave differently on 19 out of these 20 data sets; they are not sufficiently different on the data set* breast-w*. Decision tree and naïve Bayes algorithms are fundamentally different: For example, the former makes no assumptions on the data set, while the latter assumes statistical independence between attributes; the former discretizes numeric attributes, while the latter can apply density estimation on numeric attributes; the former uses special treatment for missing values, while the latter handles missing values naturally.

### 3.5. Performance

We report the values of accuracy in Table 4. The results have shown support for the idea that we could possibly obtain better classification performance by using different classification algorithms to train classifiers in an ensemble. In the table, HE is for the hybrid ensemble, and the content of a cell indicates the mean and standard deviation calculated over 20 runs of 10-fold cross-validation for applying the algorithm corresponding to the column on the data set corresponding to the row. Generally speaking, compared to a single classifier, an ensemble would show stabilized classification performance, especially when the underlying classification algorithm is an unstable one. We can see this from the fact that the standard deviations given by ensembles are lower than those given by single classifiers in most cases. For example, on the first data set,* biomed*, the standard deviation given by DT is 0.014, that given by Bagging DT is 0.009, and that given by the hybrid ensemble DT + NB is 0.007.

In some cases, such as those where data distributions are skewed, accuracy is not a good measure for classification performance evaluation, while F1-measure is a more comprehensive measure. In Table 5, we report the values of F1-measure, which is the harmonic mean of precision and recall, for data samples that belong to the minority class. A higher value of F1-measure means better classification performance. F1-measure for minority is used to evaluate how well a classification algorithm performs on data samples that belong to the minority class, which are usually the targets in most real-world machine learning applications. A classifier or an ensemble can achieve a low error rate simply by classifying all samples to the majority class or simply by ignoring minority samples on highly unbalanced data sets. In the table, similarly, HE is for the hybrid ensemble, and the content of a cell indicates the mean and standard deviation.

To assess the significance of differences in classification performance achieved by two algorithms, we perform the Wilcoxon test, a non-parametric test, whose use is recommended by Demšar [62]. We report results for the test for accuracy and F1-measure in Tables 6 and 7, respectively. In the tables, B-DT means Bagging DT, B-NB means Bagging NB, and similarly HE is for the hybrid ensemble. In the tables, the content in a cell indicates the number of data sets on which the algorithm in the corresponding row significantly wins (outperforms) and loses to (is outperformed by) the algorithm in the corresponding column. For example, in Table 6, the first cell shows that decision tree algorithm wins in accuracy naïve Bayes algorithm on 10 data sets and it loses on 9 data sets, while the difference in accuracy is not significant on 1 data set; in Table 7, the first cell shows that decision tree algorithm wins in F1-measure naïve Bayes algorithm in 18 data sets and it does not lose on a data set, while the difference in F1-measure is not significant on 2 data sets.

We can see from Table 6 that, in terms of accuracy, the number of data sets on which HE DT + NB outperforms B-DT is slightly smaller than that on which HE DT + NB is outperformed by B-DT; the number of data sets on which HE DT + NB outperforms B-NB is much larger than that on which HE DT + NB is outperformed by B-NB. We can see from Table 7 that, in terms of F1-measure, the results are similar but HE DT + NB outperforms less. The results seem to be in opposition to creating and using hybrid ensembles. On the contrary, the results show that hybrid ensembles outperform single classifiers not deterministically but probabilistically, which is also shown by our analysis. Moreover, in this paper, we intend to explain why and when hybrid ensembles are better than non-hybrid ensembles.

We propose using different classification algorithms to train more diverse classifiers in order to create better ensembles. The basic idea is to use a combination of classifiers to naturally reduce variance and use a stronger algorithm to explicitly increase classification performance. The ensemble creation process that we propose is distinguishing, because it uses fundamentally different classification algorithms to create a hybrid ensemble. For example, it uses DT, which is often with high variance (related to high instability), and NB, which is often with low variance (related to low instability or high stability). Using such a combination of classification algorithms goes against the generally accepted sense that one should only use classification algorithms with high variance in an ensemble like Bagging. We evaluate the proposed process by using a varied collection of public data sets and two metrics. Experiment results reveal that the proposed process could achieve better performance when compared to Bagging.

### 3.6. Discussion

There are 8 data sets on which the hybrid ensemble DT + NB outperforms both Bagging DT and Bagging NB:* biomed*,* breast-w*,* credit-g*,* diabetes*,* heart-c-binary*,* heart-statlog*,* hepatitis*, and* schizo*. According to Table 4, naïve Bayes algorithm performs better in accuracy than decision tree algorithm does on these data sets. Therefore, we consider that *A*_1_ is DT and *A*_2_ is NB, which is newly introduced into the ensemble and used as another generator of diversity. For these data sets, we summarize lower bounds for accuracy gain and the probability that the gain is obtained in Table 8. In the table, the second column (Acc_1_) and the third column (Acc_2_) are from the second column and the third column in Table 4, respectively; the fourth column is from the second column in Table 2; the fifth column is from the second column in Table 3; the sixth and seventh columns are lower bounds for accuracy gain and the probability that the gain is obtained, respectively, and both are calculated by (6), or (15), given that *α*_1_ is 0.05 and *δ* is 0.05; the eighth and ninth columns are from the fourth and sixth columns in Table 4, respectively; the tenth column is the accuracy difference between HE DT + NB and B-DT. According to Table 8, on all these data sets, the accuracy difference, or the actual gain given by the hybrid ensemble DT + NB against Bagging NB, is larger than the lower bound; all the probabilities are high, except the one for the data set number 3,* breast-w*, and this means that the hybrid ensemble DT + NB would highly probably outperform Bagging DT (and this indeed the case). Nevertheless, the lower bound is too loose (but it is still the only one presented so far), and finding a tighter bound would be part of the future work.

## 4. Conclusion

Ensemble learning is to train classifiers and then combine their classifications to make an overall classification. Many researchers use ensembles of classifiers created by using a single classification algorithm in various applications. These are non-hybrid ensembles, and why they work is becoming clearer. Some researchers propose the use of the mixture of two different types of classification algorithms in the creation of a hybrid ensemble. In this paper, we investigate why hybrid ensembles work, which is somewhat unclear. We present our theoretical analysis from the standpoint of diversity, which plays a significant role in ensemble learning and is one of the fundamental elements of the success of ensembles. This is the most distinguishing characteristic of this paper. We also report and discuss experiment results obtained from hybrid ensembles of classifiers created by decision tree and naïve Bayes classification algorithms, each of which is a top data mining algorithm and often used to create non-hybrid ensembles. These are two fundamentally different classification algorithms, and therefore it is interesting to see that hybrid ensembles created with them together can achieve similar or even better classification performance compared to non-hybrid ensembles created with them individually. In short, we contribute to a complement to the theoretical foundation of creating and using hybrid ensembles. The hybrid ensemble performs better not in a deterministic but a probabilistic manner. In a hybrid ensemble where two classification algorithms are used, if two are different and one is unstable while the other is more accurate, then there is a higher probability that we can have a higher value of accuracy gain; the gain is measured against an ensemble where only one of the two classification algorithms is used.

## Figures and Tables

**Algorithm 1:**
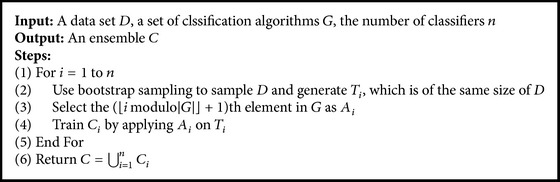
Process to create a hybrid ensemble.

**Algorithm 2:**
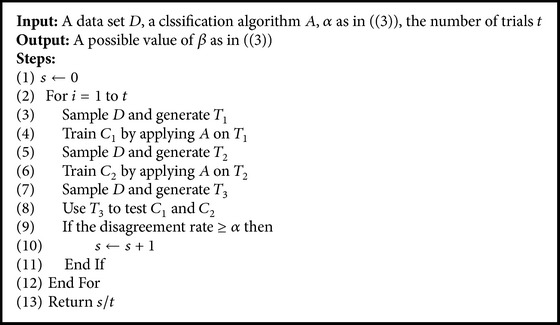
Process to estimate instability.

**Algorithm 3:**
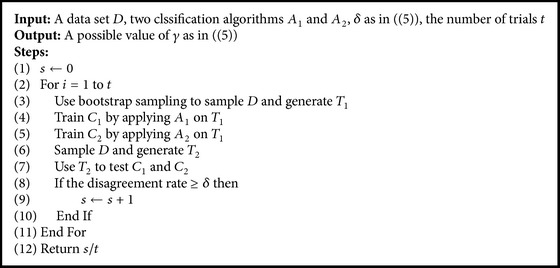
Process to estimate differentiability.

**Table 1:** Characteristics of the data sets used in experiments.

Number	Name	Samples		Attributes	
		All	Min.	Nom.	Number
(1)	biomed	209	75	1 (0)	7 (2)
(2)	boston-binary	506	132	0 (0)	13 (0)
(3)	breast-w	699	241	0 (0)	9 (1)
(4)	colic	368	136	15 (14)	7 (7)
(5)	credit-a	690	307	9 (5)	6 (2)
(6)	credit-g	1000	300	13 (0)	7 (0)
(7)	credit	490	217	9 (5)	6 (2)
(8)	diabetes	768	268	0 (0)	8 (0)
(9)	heart-c-binary	303	138	7 (1)	6 (1)
(10)	heart-h-binary	294	106	7 (5)	6 (4)
(11)	heart-statlog	270	120	0 (0)	13 (0)
(12)	hepatitis	155	32	13 (10)	6 (5)
(13)	hprice-binary	546	271	11 (0)	0 (0)
(14)	ICU	200	40	16 (0)	3 (0)
(15)	ionosphere	351	126	0 (0)	34 (0)
(16)	kr-vs-kp	3196	1527	36 (0)	0 (0)
(17)	schizo	340	163	2 (0)	11 (11)
(18)	sick	3772	231	22 (1)	7 (7)
(19)	sonar	208	97	0 (0)	60 (0)
(20)	vote	435	168	16 (16)	0 (0)

**Table 2:** Results for instability.

Number	DT	NB
(1)	0.16	0.01
(2)	0.04	0.06
(3)	0	0
(4)	0.61	0.45
(5)	0.72	0.25
(6)	1	0.97
(7)	0.61	0.23
(8)	0.96	0.66
(9)	0.9	0.37
(10)	0.9	0.18
(11)	0.7	0.33
(12)	0.72	0.58
(13)	1	0.24
(14)	0.76	0.68
(15)	0.05	0.49
(16)	0	0.18
(17)	1	0.85
(18)	0	0.04
(19)	0.19	0.9
(20)	0	0

**Table 3:** Results for differentiability.

Number	DT versus NB
(1)	0.93
(2)	1
(3)	0.05
(4)	1
(5)	1
(6)	1
(7)	1
(8)	1
(9)	1
(10)	1
(11)	1
(12)	0.98
(13)	1
(14)	0.97
(15)	1
(16)	1
(17)	1
(18)	1
(19)	1
(20)	0.95

**Table 4:** Performance in accuracy.

Number	Single		Bagging		HE
	DT	NB	DT	NB	DT + NB
(1)	0.891 ± 0.014	0.894 ± 0.005	0.908 ± 0.009	0.893 ± 0.005	0.909 ± 0.007
(2)	0.901 ± 0.008	0.709 ± 0.003	0.918 ± 0.007	0.717 ± 0.005	0.873 ± 0.006
(3)	0.948 ± 0.004	0.964 ± 0.001	0.958 ± 0.004	0.961 ± 0.001	0.967 ± 0.002
(4)	0.852 ± 0.004	0.784 ± 0.004	0.854 ± 0.005	0.786 ± 0.006	0.847 ± 0.006
(5)	0.857 ± 0.007	0.778 ± 0.003	0.862 ± 0.004	0.784 ± 0.003	0.828 ± 0.006
(6)	0.714 ± 0.007	0.751 ± 0.006	0.738 ± 0.009	0.759 ± 0.006	0.755 ± 0.008
(7)	0.865 ± 0.009	0.779 ± 0.005	0.882 ± 0.007	0.784 ± 0.005	0.836 ± 0.009
(8)	0.745 ± 0.007	0.755 ± 0.004	0.759 ± 0.008	0.756 ± 0.005	0.767 ± 0.004
(9)	0.775 ± 0.016	0.833 ± 0.005	0.787 ± 0.018	0.834 ± 0.004	0.835 ± 0.007
(10)	0.793 ± 0.016	0.843 ± 0.004	0.796 ± 0.014	0.845 ± 0.005	0.841 ± 0.006
(11)	0.784 ± 0.016	0.839 ± 0.007	0.801 ± 0.017	0.838 ± 0.006	0.847 ± 0.008
(12)	0.784 ± 0.016	0.839 ± 0.009	0.805 ± 0.019	0.842 ± 0.012	0.852 ± 0.008
(13)	0.766 ± 0.013	0.817 ± 0.003	0.783 ± 0.009	0.818 ± 0.003	0.818 ± 0.003
(14)	0.823 ± 0.013	0.808 ± 0.008	0.838 ± 0.012	0.806 ± 0.01	0.835 ± 0.011
(15)	0.891 ± 0.012	0.823 ± 0.005	0.925 ± 0.008	0.825 ± 0.007	0.882 ± 0.008
(16)	0.994 ± 0.001	0.878 ± 0.002	0.994 ± 0.001	0.878 ± 0.002	0.952 ± 0.002
(17)	0.562 ± 0.016	0.575 ± 0.004	0.595 ± 0.016	0.576 ± 0.006	0.60 ± 0.01
(18)	0.987 ± 0.001	0.928 ± 0.001	0.988 ± 0.001	0.927 ± 0.002	0.982 ± 0.001
(19)	0.737 ± 0.019	0.689 ± 0.009	0.787 ± 0.026	0.684 ± 0.019	0.728 ± 0.014
(20)	0.965 ± 0.003	0.90 ± 0.002	0.965 ± 0.004	0.90 ± 0.002	0.944 ± 0.003

**Table 5:** Performance in F1-measure.

Number	Single		Bagging		HE
	DT	NB	DT	NB	DT + NB
(1)	0.842 ± 0.019	0.833 ± 0.008	0.865 ± 0.012	0.833 ± 0.008	0.863 ± 0.011
(2)	0.805 ± 0.017	0.615 ± 0.002	0.842 ± 0.013	0.616 ± 0.005	0.778 ± 0.009
(3)	0.925 ± 0.006	0.944 ± 0.002	0.939 ± 0.006	0.944 ± 0.002	0.952 ± 0.002
(4)	0.782 ± 0.005	0.722 ± 0.006	0.786 ± 0.006	0.724 ± 0.006	0.772 ± 0.008
(5)	0.838 ± 0.007	0.705 ± 0.005	0.847 ± 0.004	0.709 ± 0.005	0.795 ± 0.008
(6)	0.458 ± 0.014	0.542 ± 0.01	0.497 ± 0.016	0.546 ± 0.012	0.516 ± 0.017
(7)	0.844 ± 0.01	0.707 ± 0.007	0.866 ± 0.009	0.716 ± 0.008	0.804 ± 0.012
(8)	0.617 ± 0.012	0.631 ± 0.006	0.636 ± 0.011	0.633 ± 0.008	0.635 ± 0.007
(9)	0.757 ± 0.017	0.813 ± 0.005	0.763 ± 0.021	0.814 ± 0.005	0.811 ± 0.008
(10)	0.692 ± 0.028	0.788 ± 0.004	0.706 ± 0.023	0.782 ± 0.006	0.776 ± 0.008
(11)	0.751 ± 0.017	0.815 ± 0.007	0.773 ± 0.018	0.814 ± 0.007	0.821 ± 0.01
(12)	0.408 ± 0.065	0.645 ± 0.02	0.439 ± 0.066	0.645 ± 0.02	0.652 ± 0.02
(13)	0.757 ± 0.014	0.809 ± 0.003	0.775 ± 0.011	0.811 ± 0.003	0.809 ± 0.003
(14)	0.423 ± 0.044	0.482 ± 0.016	0.455 ± 0.037	0.466 ± 0.015	0.476 ± 0.042
(15)	0.842 ± 0.017	0.778 ± 0.005	0.882 ± 0.013	0.783 ± 0.007	0.847 ± 0.01
(16)	0.994 ± 0.001	0.871 ± 0.002	0.994 ± 0.001	0.871 ± 0.002	0.945 ± 0.003
(17)	0.50 ± 0.027	0.505 ± 0.006	0.555 ± 0.022	0.509 ± 0.009	0.506 ± 0.016
(18)	0.894 ± 0.008	0.569 ± 0.006	0.901 ± 0.01	0.567 ± 0.005	0.842 ± 0.01
(19)	0.717 ± 0.02	0.702 ± 0.008	0.762 ± 0.03	0.701 ± 0.018	0.736 ± 0.015
(20)	0.955 ± 0.003	0.877 ± 0.002	0.955 ± 0.004	0.877 ± 0.003	0.928 ± 0.004

**Table 6:** Results for Wilcoxon test for accuracy (*W*/*L*: row versus column).

	NB	B-DT	B-NB	HE DT + NB
DT	10/9	0/16	10/9	8/11
NB		7/12	0/1	0/17
B-DT			12/7	9/8
B-NB				1/17

**Table 7:** Results for Wilcoxon test for F1-measure (*W*/*L*: row versus column).

	NB	B-DT	B-NB	HE DT + NB
DT	18/0	0/18	9/9	7/11
NB		8/11	1/2	2/13
B-DT			11/7	10/7
B-NB				2/12

**Table 8:** Lower bounds for accuracy gain and the probability.

Number	Acc_1_	Acc_2_	*β*_1_	*γ*	Acc. gain	Prob.	B-DT	HE	Acc. diff.
(1)	0.891	0.894	0.16	0.93	−7.5 × 10^−6^	0.9412	0.908	0.909	0.001
(3)	0.948	0.96	0	0.05	−0.00003	0.05	0.958	0.967	0.009
(6)	0.714	0.751	1	1	−9.25 × 10^−5^	1	0.738	0.755	0.017
(8)	0.745	0.755	0.96	1	−0.000025	1	0.759	0.767	0.008
(9)	0.775	0.833	0.9	1	−0.000145	1	0.787	0.835	0.048
(11)	0.784	0.839	0.7	1	−0.0001375	1	0.801	0.847	0.046
(12)	0.784	0.839	0.72	0.98	−0.0001375	0.9944	0.805	0.85	0.045
(17)	0.56	0.575	1	1	−3.75 × 10^−5^	1	0.595	0.6	0.005

## References

[1] Ranawana R., Palade V. (2006). Multi-classifier systems: review and a roadmap for developers. *International Journal of Hybrid Intelligent Systems*.

[2] Polikar R. (2006). Ensemble based systems in decision making. *IEEE Circuits and Systems Magazine*.

[3] Polikar R. (2007). Bootstrap-inspired techniques in computation intelligence. *IEEE Signal Processing Magazine*.

[4] Brown G. (2010). Ensemble learning. *Encyclopedia of Machine Learning*.

[5] Zhang C., Ma Y. (2012). *Ensemble machine learning*.

[6] Zhou Z.-H. (2012). *Ensemble Methods: Foundations and Algorithms*.

[7] Woźniak M., Graña M., Corchado E. (2014). A survey of multiple classifier systems as hybrid systems. *Information Fusion*.

[8] Fernández-Delgado M., Cernadas E., Barro S., Amorim D. (2014). Do we need hundreds of classifiers to solve real world classification problems?. *The Journal of Machine Learning Research*.

[9] Giacinto G., Roli F. (2001). Design of effective neural network ensembles for image classification purposes. *Image and Vision Computing*.

[10] Goh K.-S., Chang E., Cheng K.-T. SVM binary classifier ensembles for image classification.

[11] Pal M. (2008). Ensemble of support vector machines for land cover classification. *International Journal of Remote Sensing*.

[12] Merentitis A., Debes C., Heremans R. (2014). Ensemble learning in hyperspectral image classification: toward selecting a favorable bias-variance tradeoff. *IEEE Journal of Selected Topics in Applied Earth Observations and Remote Sensing*.

[13] Samat A., Du P., Liu S., Li J., Cheng L. (2014). E^2^LMs: ensemble extreme learning machines for hyperspectral image classification. *IEEE Journal of Selected Topics in Applied Earth Observations and Remote Sensing*.

[14] Han M., Liu B. (2015). Ensemble of extreme learning machine for remote sensing image classification. *Neurocomputing*.

[15] Cappelli R., Maio D., Maltoni D. (2002). A multi-classifier approach to fingerprint classification. *Pattern Analysis and Applications*.

[16] Maqsood I., Khan M. R., Abraham A. (2004). An ensemble of neural networks for weather forecasting. *Neural Computing and Applications*.

[17] Dong Y.-S., Han K.-S. A comparison of several ensemble methods for text categorization.

[18] Rohlfing T., Maurer C. R. (2005). Multi-classifier framework for atlas-based image segmentation. *Pattern Recognition Letters*.

[19] Avidan S. (2007). Ensemble tracking. *IEEE Transactions on Pattern Analysis and Machine Intelligence*.

[20] Shen H.-B., Chou K.-C. (2006). Ensemble classifier for protein fold pattern recognition. *Bioinformatics*.

[21] Cho S. B., Won H.-H. (2007). Cancer classification using ensemble of neural networks with multiple significant gene subsets. *Applied Intelligence*.

[22] Gray D., Tao H. View point invariant pedestrian recognition with an ensemble of localized features.

[23] Paisitkriangkrai S., Shen C., van den Hengel A. (2016). Pedestrian detection with spatially pooled features and structured ensemble learning. *IEEE Transactions on Pattern Analysis and Machine Intelligence*.

[24] Aljamaan H. I., Elish M. O. An empirical study of bagging and boosting ensembles for identifying faulty classes in object-oriented software.

[25] Laradji I. H., Alshayeb M., Ghouti L. (2015). Software defect prediction using ensemble learning on selected features. *Information and Software Technology*.

[26] Su Y., Shan S., Chen X., Gao W. (2009). Hierarchical ensemble of global and local classifiers for face recognition. *IEEE Transactions on Image Processing*.

[27] Katakis I., Tsoumakas G., Vlahavas I. (2010). Tracking recurring contexts using ensemble classifiers: an application to email filtering. *Knowledge and Information Systems*.

[28] Kotsiantis S., Patriarcheas K., Xenos M. (2010). A combinational incremental ensemble of classifiers as a technique for predicting students' performance in distance education. *Knowledge-Based Systems*.

[29] Takemura A., Shimizu A., Hamamoto K. (2010). Discrimination of breast tumors in ultrasonic images using an ensemble classifier based on the adaboost algorithm with feature selection. *IEEE Transactions on Medical Imaging*.

[30] Ko B. C., Gim J. W., Nam J. Y. (2011). Cell image classification based on ensemble features and random forest. *Electronics Letters*.

[31] Fraz M. M., Remagnino P., Hoppe A. (2012). An ensemble classification-based approach applied to retinal blood vessel segmentation. *IEEE Transactions on Biomedical Engineering*.

[32] Mohapatra S., Patra D., Satpathy S. (2014). An ensemble classifier system for early diagnosis of acute lymphoblastic leukemia in blood microscopic images. *Neural Computing and Applications*.

[33] Borbora Z., Srivastava J., Hsu K.-W., Williams D. Churn prediction in MMORPGs using player motivation theories and ensemble approach.

[34] Xia R., Zong C., Li S. (2011). Ensemble of feature sets and classification algorithms for sentiment classification. *Information Sciences*.

[35] Fersini E., Messina E., Pozzi F. A. (2014). Sentiment analysis: bayesian ensemble learning. *Decision Support Systems*.

[36] Wang G., Sun J., Ma J., Xu K., Gu J. (2014). Sentiment classification: the contribution of ensemble learning. *Decision Support Systems*.

[37] Hagen M., Potthast M., Büchner M., Stein B. Twitter sentiment detection via ensemble classification using averaged confidence scores.

[38] Kodovský J., Fridrich J., Holub V. (2012). Ensemble classifiers for steganalysis of digital media. *IEEE Transactions on Information Forensics and Security*.

[39] Singh K. P., Gupta S., Rai P. (2013). Identifying pollution sources and predicting urban air quality using ensemble learning methods. *Atmospheric Environment*.

[40] Govindarajan M. (2014). Hybrid intrusion detection using ensemble of classification methods. *International Journal of Computer Network and Information Security*.

[41] Doan H. T. X., Foody G. M. (2007). Increasing soft classification accuracy through the use of an ensemble of classifiers. *International Journal of Remote Sensing*.

[42] Kuncheva L. I., Skurichina M., Duin R. P. W. (2002). An experimental study on diversity for bagging and boosting with linear classifiers. *Information Fusion*.

[43] Langdon W. B., Barrett S. J., Buxton B. F. Combining decision trees and neural networks for drug discovery.

[44] Lu Z., Wu X., Bongard J. Adaptive informative sampling for active learning.

[45] Hsu K.-W. Hybrid ensembles of decision trees and artificial neural networks.

[46] Domingos P. A unified bias-variance decomposition for zero-one and squared loss.

[47] Cortes C., Vapnik V. (1995). Support-vector networks. *Machine Learning*.

[48] Wu X., Kumar V., Quinlan J. R. (2008). Top 10 algorithms in data mining. *Knowledge and Information Systems*.

[49] Salgado R. M., Pereira J. J. F., Ohishi T., Ballini R., Lima C. A. M., Von Zuben F. J. A hybrid ensemble model applied to the short-term load forecasting problem.

[50] Min J.-K., Cho S.-B. Activity recognition based on wearable sensors using selection/fusion hybrid ensemble.

[51] Verikas A., Kalsyte Z., Bacauskiene M., Gelzinis A. (2010). Hybrid and ensemble-based soft computing techniques in bankruptcy prediction: a survey. *Soft Computing*.

[52] Verma B., Hassan S. Z. (2011). Hybrid ensemble approach for classification. *Applied Intelligence*.

[53] Breiman L. (1996). Bagging predictors. *Machine Learning*.

[54] Quinlan J. R. (1993). *C4.5: Programs for Machine Learning*.

[55] Opitz D., Maclin R. (1999). Popular ensemble methods: an empirical study. *Journal of Artificial Intelligence Research*.

[56] Kuncheva L. I. (2005). Using diversity measures for generating error-correcting output codes in classifier ensembles. *Pattern Recognition Letters*.

[57] Hsu K.-W., Srivastava J. Diversity in combinations of heterogeneous classifiers.

[58] Hsu K.-W., Srivastava J. Relationship between diversity and correlation in multi-classifier systems.

[59] Hsu K.-W., Srivastava J. (2012). Improving bagging performance through multi-algorithm ensembles. *Frontiers in Computer Science*.

[60] Lichman M. (2013). *UCI Machine Learning Repository*.

[61] Hall M., Frank E., Holmes G., Pfahringer B., Reutemann P., Witten I. H. (2009). The WEKA data mining software: an update. *ACM SIGKDD Explorations Newsletter*.

[62] Demšar J. (2006). Statistical comparisons of classifiers over multiple data sets. *The Journal of Machine Learning Research*.

